# Earliest Human Presence in North America Dated to the Last Glacial Maximum: New Radiocarbon Dates from Bluefish Caves, Canada

**DOI:** 10.1371/journal.pone.0169486

**Published:** 2017-01-06

**Authors:** Lauriane Bourgeon, Ariane Burke, Thomas Higham

**Affiliations:** 1 Département d'Anthropologie, Université de Montréal, Montréal QC, Canada; 2 Oxford Radiocarbon Accelerator Unit, Research Laboratory for Archaeology & the History of Art, University of Oxford, Oxford, United Kingdom; New York State Museum, UNITED STATES

## Abstract

The timing of the first entry of humans into North America is still hotly debated within the scientific community. Excavations conducted at Bluefish Caves (Yukon Territory) from 1977 to 1987 yielded a series of radiocarbon dates that led archaeologists to propose that the initial dispersal of human groups into Eastern Beringia (Alaska and the Yukon Territory) occurred during the Last Glacial Maximum (LGM). This hypothesis proved highly controversial in the absence of other sites of similar age and concerns about the stratigraphy and anthropogenic signature of the bone assemblages that yielded the dates. The weight of the available archaeological evidence suggests that the first peopling of North America occurred ca. 14,000 cal BP (calibrated years Before Present), i.e., well after the LGM. Here, we report new AMS radiocarbon dates obtained on cut-marked bone samples identified during a comprehensive taphonomic analysis of the Bluefish Caves fauna. Our results demonstrate that humans occupied the site as early as 24,000 cal BP (19,650 ± 130 ^14^C BP). In addition to proving that Bluefish Caves is the oldest known archaeological site in North America, the results offer archaeological support for the “Beringian standstill hypothesis”, which proposes that a genetically isolated human population persisted in Beringia during the LGM and dispersed from there to North and South America during the post-LGM period.

## Introduction

Beringia, a vast region stretching from the Lena River in Siberia to the Mackenzie River in the Yukon Territory [1, 2], is thought to have played a pivotal role in the initial dispersal of human populations from Asia to North America. The exact timing of the initial dispersal remains uncertain, however. Recent genetic and palaeogenetic analyses [3–10], as well as dental morphological evidence [11], confirm that human populations migrating into North America originated in Siberia. They also suggest that dispersing groups reached Beringia during the LGM (dated to ca. 18,000–24,000 cal BP) where they were genetically isolated for up to 8,000 years before moving south of the ice-sheets into North America [3–11]. Unfortunately, archaeological support for the standstill hypothesis is scarce [12]. Recent archaeological discoveries prove that humans were able to adapt to high-latitude, Arctic environments by at least 45,000 cal BP [13]. The Yana River sites, in Siberia, demonstrate that modern human populations had reached Western Beringia by 32,000 cal BP [14, 15], i.e., well before the LGM. Human activity is not recorded again in Western Beringia until the post-LGM period, however, with occupations of two open-air sites, Berelekh and Ushki, dated to ca. 14–13,000 cal BP [16–18]. In Eastern Beringia, the oldest currently accepted human occupations occur in the Tanana valley (interior Alaska) at Swan Point, Broken Mammoth and Mead [19–21], and at the Little John site, located 2 km east of the international border in the Yukon Territory [22]; these sites are no older than ca. 14,000 cal BP, however [19–22]. The only potential candidate for an earlier, LGM occupation of Beringia is the controversial Bluefish Caves site.

Excavated from 1977 to 1987 under the direction of Jacques Cinq-Mars (Archaeological Survey of Canada), the Bluefish Caves site (northern Yukon Territory, 67°09’N 140°45’W) occupies a unique place in Eastern Beringian prehistory. The site is comprised of three small karstic cavities, not exceeding 30 m^3^ in volume, located in the Keele range about 54 kilometres southwest of Old Crow village. The caves are situated at the base of a limestone ridge about 250 meters above the right bank of the Bluefish River [23–27]. All three cavities contain a loess layer (Unit B) up to one meter thick, deposited on bedrock (Unit A) and overlain by a humus layer mixed with cryoclastic debris (Unit C) and finally, a modern humus layer (Unit D) [25, 27]. The loess deposit (Unit B) can be differentiated into three sub-layers based on granulometric and sedimentological examinations and was excavated in 5 cm spits [23]. Small artefact series were excavated from the loess in Cave I (MgVo-1) and Cave II (MgVo-2) and rich faunal assemblages were recovered from all three caves [23–27]. The lithic assemblages (which number about one hundred specimens) include microblades, microblade cores, burins and burin spalls as well as small flakes and other lithic debris [23–26]. Most of the artefacts were recovered from the loess of Cave II at a depth comprised between about 30 to 155 cm. The deepest diagnostic pieces–a microblade core (B3.3.17), a burin (B3.6.1) and a core tablet (B4.16.4) found inside Cave II, as well as a microblade (E3.3.2) found near the cave entrance–derive from the basal loess at a depth of about 110 to 154 cm below datum, according to the CMH archives [28]. While the artefacts cannot be dated with precision [24, 25, 29], they are typologically similar to the Dyuktai culture which appears in Eastern Siberia about 16–15,000 cal BP, or possibly earlier, ca. 22–20,000 cal BP [30]. There are no reported hearth features [24]. Palaeoenvironmental evidence, including evidence of herbaceous tundra vegetation [31, 32] and vertebrate fauna typical of Pleistocene deposits found elsewhere in Eastern Beringia [27, 33, 34], is consistent with previously obtained radiocarbon dates which suggest that the loess layer was deposited between 10,000 and 25,000 ^14^C BP (radiocarbon years Before Present), i.e., between 11,000 and 30,000 cal BP [23–27, 35] (Table 1).

**Table 1:** Previous radiocarbon dates obtained on bone from Bluefish Caves I (MgVo-1) and II (MgVo-2).

Lab number	Age ^14^C BP	Specimen dated	Ref.
**MgVo-1**			
CAMS-23468	12830 ± 60	Caribou radius	^b^
CAMS-23472	11570 ± 60	Moose metacarpal	^b^
CAMS-23473	13580 ± 80	cf. Dall sheep tibia	^b^
CRNL-1220	12845 ± 250	Mammoth tibia	^b^
GSC-2881^a^	12900 ± 100	Horse femur	[23]
RIDDL-277	12210 ± 210	Caribou metatarsal	^b^
RIDDL-278	17440 ± 220	Horse metatarsal	^b^
RIDDL-559	13940 ± 160	Mammoth humerus	^b^
**MgVo-2**			
Beta-126870	7780 ± 60	Snow goose scapula	^b^
Beta-140679	21100 ± 150	Caribou or sheep	^b^
CAMS-23469	31730 ± 230	Bison tibia	^b^
CAMS-23470	22740 ± 90	Mammoth limb bone	^b^
CRNL-1221	17880 ± 330	Mammoth scapula	^b^
CRNL-1237	22680 ± 530	Horse limb bone	[35]
GSC-3053^a^	15500 ± 130	Mammoth scapula	[26]
RIDDL-223	20230 ± 180	Mammoth scapula	^b^
RIDDL-224	23910 ± 200	Mammoth limb bone	^b^
RIDDL-225	23200 ± 250	Mammoth limb bone	^b^
RIDDL-226	24820 ± 115	Caribou tibia	^b^
RIDDL-330	19640 ± 170	Mammoth scapula	^b^
RIDDL-561	10230 ± 140	cf. Bison metacarpal	^b^

Laboratory identification: GSC, Geological Survey of Canada, Earth Sciences section of Natural Resources Canada, Ontario; CRNL, Chalk River Nuclear Laboratories, Anatomic Energy of Canada Ltd., Ontario; RIDDL, RadioIsotope Direct Detection Laboratory, Simon Fraser University, British-Columbia; Beta, Beta Analytic, Florida; CAMS, Center for Accelerator Mass Spectrometry, Lawrence Livermore National Laboratory, California.

^a^ Conventional dates. All other dates are AMS measurements.

^b^ Unpublished dates, only available online at http://card.anth.ubc.ca/.

Chronological evidence from Bluefish Caves I and II led to the initial suggestion that human occupation of Eastern Beringia occurred before the LGM, as early as 24,800 ^14^C BP [24–27]. Doubts as to the stratigraphic integrity of the site and anthropogenic nature of the bone samples submitted for radiocarbon analysis did not encourage the scientific community to accept this hypothesis, unfortunately [29, 36–38]. In order to clarify the nature of the bone assemblages and to establish the chronology of human occupation of the site, we undertook a re-analysis of the faunal assemblages from Bluefish Caves I and II from a rigorous, taphonomic perspective. In a recent article published by one of us [39], we presented a study of the faunal assemblage from Cave II and showed that humans partially contributed to the modification of the bone material. Here, we extend our analysis to the faunal assemblage of Cave I, focus on identifying undisputable traces of human activity and provide new, accurate and reliable AMS dates on cut-marked bone specimens from both caves.

## Materials and Methods

### Taphonomic analyses

The faunal collections from Bluefish Caves I and II are curated at the Canadian Museum of History (Gatineau, QC). Taxonomic and anatomical identifications were made using the “Pierrard et Bisaillon” faunal collection, housed at the Zooarchaeology laboratory of the Université de Montréal, and taphonomic analyses were conducted in the Ecomorphology and Paleoanthropology laboratory (U. de Montréal).

A full taphonomic analysis is required in order to contextualise and thus correctly identify culturally modified bone. The sedimentary and geological context of an archaeological site affects the taphonomic signature of the faunal assemblages it contains. As previously stated, the faunal material from Bluefish Cave derives from loess, i.e., fine particles of aeolian silt which should not produce scratches on bones but can lead to polished surfaces [40, 41]. Cryoclastic debris incorporated into the loess [23–27], however, may have abraded the bone surfaces. Rockfall can also modify bone, producing striations and patterns of bone breakage [41–44]. In some cases, these natural traces can mimic cut marks [43–46], raising the spectre of equifinality. This has led some researchers, in the past, to question the cultural origin of some of the bone modifications reported in the literature [see for example refs. 36 and 45].

Other agents are capable of creating patterns of bone modification that can be difficult to distinguish from human activity. This is particularly true of large canids, which are capable of applying static pressure on bone resulting in the production of spiral bone fractures and bone flakes similar to the ones produced by human marrow extraction [41, 47]. Carnivore activity is also usually accompanied by the presence of digested bone, as well as pits, punctures, scoring and furrows altering the bone surface [48–51]. Carnivore teeth produce traces with characteristically “U” shaped profiles when viewed in cross-section and which are wider and shallower than cut marks, making them easily distinguishable from human modifications (see below) [44, 51, 52]. The frequency of tooth marks, spiral fractures and bone flakes were recorded; tooth marks were noted as “certain” or “probable” when the identification couldn’t be confidently assessed.

The climatic context of a site is also important because weathering and freeze-thaw cycles can lead to cracks and desquamation of bone surfaces [53–55], potentially removing or altering traces of cultural activities. Damage due to climatic factors (e.g., weathering) but also due to biological agents (e.g., root etching, trampling and rodent gnawing) were carefully recorded and quantified, therefore, in order to control for possible loss of information.

We conducted initial taphonomic analyses under reflected light using an Olympus SZ61 stereomicroscope at low magnification (6.7 – 45x). Bone alterations resulting from natural processes cited above were recorded and specimens bearing potential traces of cultural modification were selected for further analysis based on a combination of morphological and morphometrical criteria.

Several morphological features can be used to distinguish cut marks made with stone tools from traces produced by natural processes [43–46, 48, 52, 56–58] and the following criteria were noted for each potential cut mark:

1. Shape: cut marks made with stone tools are usually V-shaped (narrow \/ or wide \_/) while carnivore tooth marks or even metal tools will produce grooves with more parallel walls (U or |_|) [44–46, 52, 56–58]. The cross-sectional shape of a cut mark can be symmetrical (\/) or asymmetrical (√) depending on the inclination of the tool relative to the bone surface [57, 59]. Morphometrical analyses allow us to quantify the profile of potential cut marks by measuring the breadth at the top and at the bottom of the groove (see below);
2. Trajectory: cut marks are commonly straight but can sometimes be curved; they are rarely sinuous, as in the case of trampling or root etching [44–46];
3. Number of striae, size and overlapping: butchering activities can produce multiple striae that should be parallel in orientation and roughly the same size [45]; a scraping motion to remove meat may produce overlapping striae [45, 46]; trampling marks will not be oriented in this fashion [45, 46].
4. Shoulder effect and shoulder flaking: shallow striations along the main groove and edge flaking can sometimes be observed under the microscope at high magnifications in marks produced by butchering; they are rarer in marks created by trampling [44–46];
5. Internal microstriations: microstriations are common on the inner walls of marks produced by stone tools during butchery; they may also be produced by trampling but will not be observed in cases of root etching or tooth mark scoring [44, 46].
6. Anatomical location and orientation: the anatomical location and orientation of cultural bone modifications must be consistent with the marks produced by specific butchery tasks described in the literature [48, 60–62]; marks produced by natural processes, however, will not reflect any predetermined intention [43]. Assigning a precise function to a cut mark is subject to equifinality since marks resulting from skinning, defleshing and dismembering can occur in very similar locations and because variability exists in the placement and orientation of butchery marks [56]. Keeping this in mind, we noted the anatomical location and orientation of each cut mark and we proposed a potential butchery task based on comparisons with ethnozooarchaeological data [48, 60–62].

We undertook morphometrical analyses of potential cultural modifications using the Olympus DSX-100 microscope, equipped with a motorised stage (16x optical zoom). High resolution digital images of the median cross-section of suspected cut marks were captured and the depth, breadth and opening angle were measured using integrated tools. While these measurements are often used to describe the size of cut marks [46, 52, 57–59, 63, 64], the depth and breadth of a mark can greatly vary depending on the butchery action, carcass size or hardness of the bone surface [57, 58, 63]. Furthermore, the opening angle of a cut mark varies according to the attrition of the tool edge [59, 64]. The breadth ratio (the ratio between the breadth at the top and the breadth at the bottom of the cut mark) [58] better illustrates the shape of the groove (\/ or |_|) and is a good criterion for distinguishing between cut marks made with stone tools and modifications produced by other effectors [44–46, 52, 58]. Small ratios are associated with grooves with parallel walls while large ratios are associated with narrow \/ or wide \_/-shaped grooves such as the ones produced by stone tools [52, 58]. Therefore, we calculated the breadth ratio for each potential cut mark and compared the values obtained to the ranges reported for experimental and archaeological data for a variety of tools [58] and carnivore tooth marks [52].

In summary, in order for a bone modification to be identified as a cut mark in this study, all of the above criteria had to be met. If one of the criteria couldn’t be confidently assessed, the mark was consigned to a “probable” category of human bone modification.

We selected six bone samples bearing indisputable evidence of butchery activity for radiocarbon dating. The samples were sent to the Oxford Radiocarbon Accelerator Unit (ORAU) for analysis.

### Radiocarbon dating

ORAU dated the samples using an Accelerator Mass Spectrometry (AMS) and the following chemical pretreatment protocol:

- Coarsely ground bone powder was loaded into a glass test tube baked out at 500°C prior to use;
- A sequence of 0.5M HCl, 0.1M NaOH and 0.5M HCl was used to treat the bone, interspersed with rinsing with ultra-pure (MilliQ™) water between each reagent;
- Crude collagen was gelatinised in pH3 solution at 75°C for 20 hours;
- The gelatin solution was filtered using a polyethylene Eezi-filter™ whose pore size ranges between 45–90 μm, that is precleaned by thorough rinsing and ultrasonication and the insoluble residues discarded;
- The filtered gelatin was then pipetted into a precleaned ultra-filter (Sartorius 'Vivaspin Turbo 15' ultrafilters (VS15T22) with a 30kD MWCO) and centrifuged at 2500–3000 rpm until 0.5–1 mL of the >30 kD gelatin fraction remains (typically 20–40 min) (for the human bone this was not applied due to the low sample size of the recovered collagen);
- This gelatin was freeze-dried ready for combustion in a CHN analyser.

The ultrafiltration step was originally described by Brown and colleagues [65]. Precleaning steps are undertaken using the protocols outlined by Brock and colleagues [66].

Combusted gelatin samples were analysed using a PDZ-Europa Robo-Prep biological sample converter (combustion elemental analyser) coupled to a PDZ-Europa 20/20 mass spectrometer operating in continuous flow mode using an He carrier gas. This enables the measurement of both δ^15^N and δ^13^C isotope ratios, nitrogen and carbon content as well as the C:N atomic ratios. VPDB ισ the standard for δ^13^C values. Graphite was produced by reacting the sample CO_2_ over an iron catalyst in an excess H_2_ atmosphere at 560°C. AMS radiocarbon measurement was carried out using the ORAU 2.5MV HVEE accelerator. All bones were well preserved in terms of collagen and had C:N atomic ratios of 3.2, as expected for intact collagen. We used OxCal 4.2 [67] and the INTCAL113 calibration curve [68] to calibrate the radiocarbon data.

## Results

A total of 36,000 mammal bones from Caves I and II were analysed for this research. As previously reported [23–27], the faunal spectrum of the Bluefish Caves is diversified and includes several carnivore taxa. Our taphonomic analysis indicates that wolves, lions and, to a lesser degree, foxes were the main agents of bone accumulation and modification, but that humans also contributed to the bone accumulations in both caves, partially confirming earlier taphonomic studies [26, 39]. The caves were probably used as den sites for Ursids in winter and Canids in spring and summer. The archaeozoological evidence, together with the small size of the lithic assemblages, suggests that human occupation of Caves I and II was probably sporadic and brief.

The effects of weathering and cryoturbation were observed in both caves but didn’t alter the bone surfaces significantly [39]. In Cave II, however, the extent of root etching on the bone surfaces was very high and will have affected the preservation of cut marks: root etching was recorded on 70% of the bone material in this cavity and affected more than half of the surface in 27% of cases. The bone assemblage of Cave I was less affected by this biological agent with only 4% of the bones affected. On the other hand, 57% of the bones in Cave I and only 18% in Cave II were abraded. Rodent gnawing was minimally present, while carnivore tooth marks were observed on 38 to 56% of the bone material of Cave I and 14 to 32% of the material of Cave II. Thus, carnivore gnawing, root etching and abrasion were mainly responsible for the bone alterations in Cave I and II (see also S1 Graph). In both cavities, however, human modifications were also identified.

**Fig 1:**
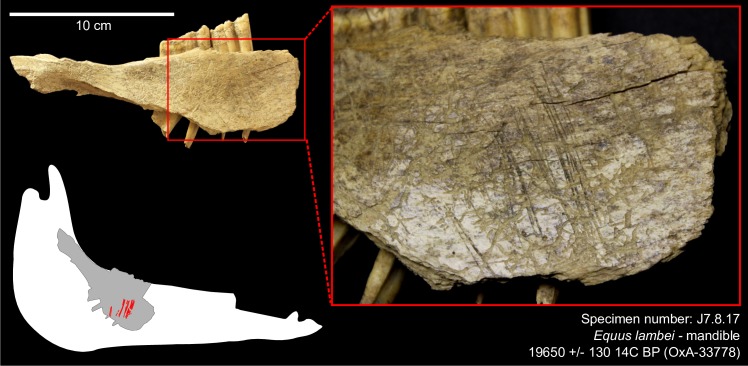
Cut marks on a horse mandible from Cave II. The specimen (# J7.8.17) is dated to 19,650 ± 130 ^14^C BP (OxA-33778). The bone surface is a bit weathered and altered by root etching but the cut marks are well preserved; they are located on the medial side, under the third and second molars, and are associated with the removal of the tongue using a stone tool [48].

**Fig 2:**
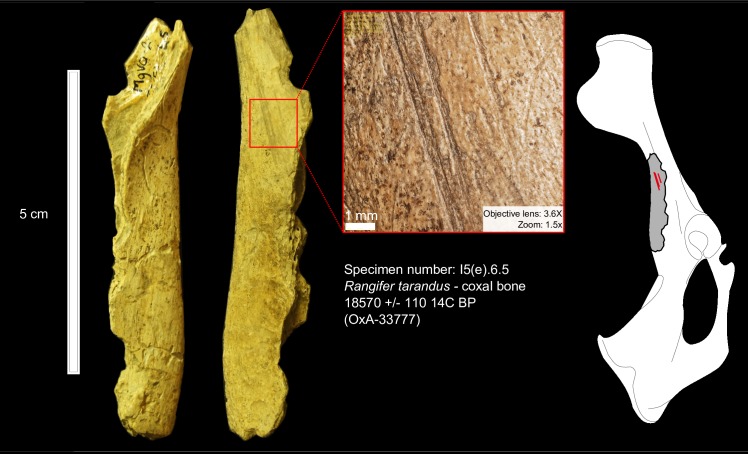
Cut marks on a caribou coxal bone from Cave II. The specimen (# I5.6.5) is dated to 18,570 ± 110 ^14^C BP (OxA-33777) and shows straight and parallel marks resulting from filleting activity.

We recorded a total of fifteen bone samples with cultural modifications confidently attributable to human activities (N = 10 in Cave I and N = 5 in Cave II), based on morphological and morphometrical criteria, and twenty more samples with “probable” cultural modifications for a total of less than 1% of the faunal remains. The fifteen cut-marked specimens are illustrated and described in the Supporting Information files (S1 Table, S2 Table, S2 Graph, S3 Table, S1 Fig). Different activities are attested including skinning, dismembering and defleshing. Cut marks were observed on horse (*Equus lambei*) (Fig 1), caribou (*Rangifer tarandus*) (Fig 2), wapiti (*Cervus elaphus*), and possibly Dall sheep (*Ovis dalli*) and bison (*Bison priscus*), and include a previously published bird scapula [69].

**Fig 3:**
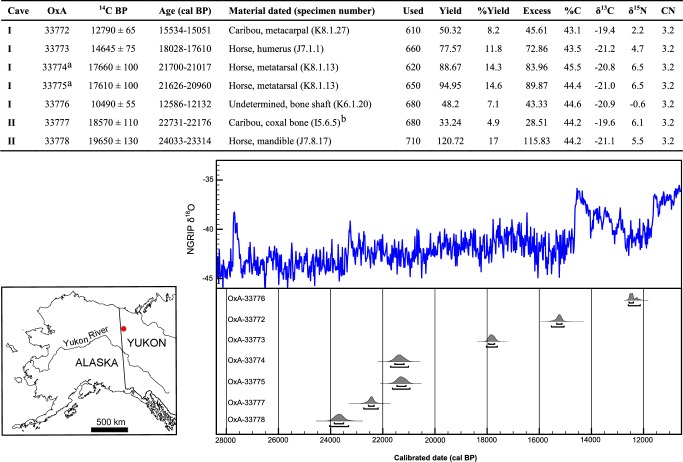
New radiocarbon determinations obtained on bone from Bluefish Caves I and II. The red dot on the map indicates the location of the site. Conventional radiocarbon ages are expressed in ^14^C BP [70], values are ± one standard error [71]. Calibrations (cal BP) were made using OxCal 4.2 [67] and the INTCAL113 calibration curve [68]. ‘Used’ represents the amount of bone powder pretreated in milligrams. ‘Yield’ represents the weight of collagen or ultrafiltered collagen in milligrams. ‘%Yield’ is the percent yield of extracted collagen as a function of the starting weight of the bone analyzed. ‘%C’ is the carbon present in the combusted collagen and averages 40 ± 2% in the ORAU. Stable isotope ratios are expressed in ‰ relative to vPDB [72] with a mass spectrometric precision of ± 0.2 ‰ for C and ± 0.3 ‰ for N. ‘CN’ is the atomic ratio of C to N and is acceptable if it ranges between 2.9–3.5. ^a^Denote duplicate measurements from the start of the pretreatment chemistry. ^b^Specimen also identified in previous article [39].

Six of the cut-marked bones were selected for AMS dating (Fig 3). The results range from 10,490 ± 55 ^14^C BP to 19,650 ± 130 ^14^C BP, i.e., between 12,000 and 24,000 cal BP, and are consistent with previously reported dates for Bluefish Caves (Table 1) [23–27, 35]. An old date that was obtained by the RadioIsotope Direct Detection Laboratory on a cut-marked horse metatarsal from Cave I (17,440 ± 220 ^14^C BP; RIDDL-278) is now strengthened by two new dates performed on the same specimen (Figure D in S1 Fig): 17,660 ± 100 ^14^C BP (OxA-33774) and 17,610 ± 100 ^14^C BP (OxA-33775).

The oldest date we obtained (19,650 ± 130 ^14^C BP, OxA-33778) came from the cut-marked horse mandible (J7.8.17) from Cave II (Fig 1) and is consistent with the stratigraphic position of the bone, which is reported to have been found in the basal loess, at a depth of 142 cm below datum [28]. Unfortunately, the exact depth at which the other bone specimens we dated in this study were found could not be established from the archival records at our disposal, but the vast majority of the faunal material derives from the bone beds contained in the lower loess layers in both caves [28].

It is highly unlikely that the cut marks observed on the Bluefish Caves faunal material were generated by nonhuman agents or natural processes. In Cave II, the horse mandible (J7.8.17) (Fig 1) and a caribou pelvis (I5.6.5) (Fig 2) date the human presence to the LGM, ca. 24–22,000 cal BP (Fig 3). The traces identified on these bones are clearly not the result of climato-edaphic factors or carnivore activity. The presence of multiple, straight and parallel marks with internal microstriations observed on both specimens eliminates carnivores as potential agents. The relatively high breadth ratio (12 and 18 μm, respectively), as well as the depth (91 and 95 μm, respectively) and opening angle (144 and 139 μm, respectively) that we measured are in the range of marks produced by stone tools reported by experimental and archaeological studies [58]; the breadth ratio also differs from marks produced by carnivore teeth [52]. Sedimentary abrasion or trampling are also eliminated since the caribou coxal bone shows no other signs of abrasion and the long, parallel striae on the horse mandible are simply too regular. Furthermore, the anatomical location and orientation of the marks are consistent with filleting marks in the case of the caribou bone, while the presence of multiple cut marks on the medial side of the horse mandible indicates the removal of the tongue [48]. Previous cementochronological analysis of one of the teeth from this mandible indicated that the animal was killed in spring/summer [35], thus suggesting a human presence in Cave II during the warm season.

In Cave I, three other bone specimens are dated to the end of the Pleistocene, ca. 22–15,000 cal BP. A horse humerus (J7.1.1), a horse metatarsal (K8.1.13) and a caribou metacarpal (K8.1.27) bear straight, V-shaped cut marks on the shaft (no more than two on each bone) that cannot result from natural processes; again, morphometrical analyses show consistency with marks produced by stone tools [52, 58] and the relatively deep and narrow incisions can hardly result from trampling [44]. Instead, the marks observed on the humerus and metacarpal indicate filleting activity (Figures C and H in S1 Fig) while the cut-marked metatarsal may reflect stripping of tendons [48] (Figure D in S1 Fig).

Finally, the youngest date (ca. 12,000 cal BP) was obtained on a bone fragment (K6.1.20) from Cave I, bearing eight clear, curved and parallel striae obliquely oriented on the bone shaft and illustrating filleting activity. The specimen couldn’t be taxonomically identified but might be a wapiti bone judging by the thickness and curvature of the shaft fragment (Figure F in S1 Fig). Cut marks were also observed on a wapiti premaxilla (undated) recovered from the same cavity and reflecting skinning activity (Figure A in S1 Fig).

Thus our recently obtained AMS dates confirm that Bluefish Caves is the oldest known archaeological site in North America and indicate that people used the caves on several occasions over a relatively long time, spanning from the cold period of the LGM to the Pleistocene/Holocene transition.

## Discussion

The small percentage of cut-marked bones at Bluefish Caves I and II is not surprising. Our taphonomic analysis suggests that natural processes, particularly root etching and scavenging activities, may have destroyed some of the evidence of human activity. In other Beringian archaeological sites dated to the Late Pleistocene/Early Holocene, taphonomic studies show that cut marks are scarce (less than 1%) [73] or even absent in bone assemblages highly affected by natural processes [74, 75]. Furthermore, the Bluefish Caves, like other Beringian cave sites [73, 74, 76], were probably only used occasionally as short-term hunting sites. Thus, they differ from the open-air sites of the Tanana valley in interior Alaska and the Little John site in the Yukon Territory, where hearth features, large lithic collections, bone tools and animal butchery have been identified, reflecting different cultural activities and a relatively longer-term, seasonal occupation [19–22].

In conclusion, while the Yana River sites indicate a human presence in Western Beringia ca. 32,000 cal BP [14, 15], the Bluefish Caves site proves that people were in Eastern Beringia during the LGM, by at least 24,000 cal BP, thus providing long-awaited archaeological support for the “Beringian standstill hypothesis”. According to this hypothesis, a human population genetically isolated existed in Beringia from about 15,000 to 23,000 cal BP [9], or possibly earlier [3–5, 10], before dispersing into North and eventually South America after the LGM [3–12,77–79].

Central Beringia may have sustained human populations during the LGM since it offered relatively humid, warmer conditions and the presence of woody shrubs and occasional trees that could be used for fuel [12, 33, 37]. However, this putative core region was submerged at the end of the Pleistocene by rising sea levels making data collection difficult. Bluefish Caves, situated in Eastern Beringia, may have been located at the easternmost extent of the standstill population’s geographical range. The seasonal movements of human hunters from a core range, hypothetically located in Central Beringia, into adjoining, more steppic regions such as Eastern Beringia would explain the sporadic nature of the occupations at Bluefish Caves [12].

The scarcity of archaeological evidence for LGM occupations in both Western and Eastern Beringia suggests that the standstill population was very small. This is consistent with the genetic data, which suggest that the effective female population was only about 1000–2000 individuals [5, 6, 10] and that the standstill population didn’t exceed a few tens of thousands of people in Beringia [10]. The size of the standstill population is thought to have increased after the LGM, leading to renewed dispersals into the Americas [4–6, 10]. Our results indicate that human hunters continued to use Bluefish Caves as the climate improved. While some prey species became extinct by ca. 14,000 cal BP (e.g. horse) [80], human hunters could continue to rely on different species such as caribou, bison and wapiti.

By around 15–14,000 cal BP an ice-free corridor formed between the Laurentide and Cordilleran ice sheets potentially allowing humans to disperse from Beringia to continental North America; arguably, this corridor wouldn’t have been biologically viable for human migration before ca. 13–12,500 cal BP, however [77–79]. It is now more widely recognized that the first inhabitants of Beringia probably dispersed along a Pacific coastal route, possibly as early as ca. 16,000 cal BP, and settled south of the ice sheets before the ice-free corridor became a viable route [3–12,77–79].

Our results, therefore, confirm that Bluefish Caves is the oldest known archaeological site in North America and furthermore, lend support to the standstill hypothesis. More research effort is required in Beringia clearly, to substantiate the existence of a standstill population and fully understand the prehistory of the first people of the Americas.

## Supporting Information

S1 Fig Cut-marked bone specimens from Bluefish Cave I (A-J) and Cave II (K-M). The faunal collections from Bluefish Caves are curated at the Canadian Museum of History (Gatineau, QC). Taphonomic analyses were conducted in the Ecomorphology and Paleoanthropology laboratory (U. de Montréal) and high resolution digital images were taken using the Olympus DSX-100 microscope.
(DOCX)

S1 Graph Impact of the natural taphonomic processes affecting the bone assemblages. Our taphonomic analysis began with the faunal material of Bluefish Cave II and was applied to each specimen greater than 20 mm in length (N = 5980) [see also ref. 35]. Because of time constraints, the same methodology was applied to the bone specimens of Cave I measuring more than 30 mm in length (N = 5425).
(DOCX)

S2 Graph Morphometrical analysis. Measurements obtained on fourteen cut-marked bone specimens from Bluefish Caves I and II (see S2 Table) are compared to the ranges reported for experimental and archaeological data: (1) carnivore tooth marks [48], (2) experimental steel blade, (3) archaeological data from an Italian site dated to the Iron Age (i.e. Trebbio), (4) experimental flint flakes, (5) experimental retouched tool, (6) archaeological data from an Italian site dated to the Paleolithic (i.e. Paglicci) [52], (7) cut marks from Bluefish Caves. Our graphs show that the depth and opening angle we measured are in the range reported by comparative studies [48, 52]. The breadth ratio (the ratio between the breadth at the top and the breadth at the bottom of the cut mark) is a better criterion for distinguishing between cut marks made with stone tools and modifications produced by other effectors [48, 52]. Here, we show that the relatively high breadth ratios that we obtained from Bluefish Caves are comparable to the measures obtained on cut marks produced by flint flakes and retouched tool and indicate the presence of “V-shaped” grooves [52].
(DOCX)

S1 Table Morphological analysis. Morphological features described in the article were noted for each cut mark observed on the bone specimens from Bluefish Caves I and II.
(DOCX)

S2 Table Morphometrical analysis. The depth, breadth and opening angle were measured on fourteen bone specimens from Bluefish Caves I and II bearing cultural modifications confidently attributable to human activities. Measurements were obtained on the median cross-section of cut marks [as described in ref. 52], using the Olympus DSX-100 microscope (16x optical zoom; objective lens: 3.6X). When the bone surface was too altered or when multiple incisions were present, a second measure was taken.
(DOCX)

S3 Table Ethnographic comparisons. Descriptions of the cut marks observed on the bone specimens from Bluefish Caves I and II and comparisons with ethnographic data.
(DOCX)

## References

[1] Hopkins DM, Matthews JV, Schweger CE, Young SB. Paleoecology of Beringia. New York: Academic Press; 1982. 489 p.

[2] West FH. American beginnings: the prehistory and palaeoecology of Beringia: University of Chicago Press; 1996. 576 p.

[3] Tamm E, Kivisild T, Reidla M, Metspalu M, Smith DG, Mulligan CJ, et al Beringian standstill and spread of Native American founders. PLoS One. 2007;2(9):e829 10.1371/journal.pone.0000829 17786201 PMC1952074

[4] Kitchen A, Miyamoto MM, Mulligan CJ. A three-stage colonization model for the peopling of the Americas. PLoS One. 2008;3(2):e1596 10.1371/journal.pone.0001596 18270583 PMC2223069

[5] Mulligan CJ, Kitchen A, Miyamoto MM. Updated three-stage model for the peopling of the Americas. PLoS One. 2008;3(9):e3199 10.1371/journal.pone.0003199 18797500 PMC2527656

[6] Fagundes NJ, Kanitz R, Eckert R, Valls AC, Bogo MR, Salzano FM, et al Mitochondrial population genomics supports a single pre-Clovis origin with a coastal route for the peopling of the Americas. The American Journal of Human Genetics. 2008;82(3):583–92. 10.1016/j.ajhg.2007.11.013 18313026 PMC2427228

[7] Achilli A, Perego UA, Lancioni H, Olivieri A, Gandini F, Kashani BH, et al Reconciling migration models to the Americas with the variation of North American native mitogenomes. Proceedings of the National Academy of Sciences. 2013;110(35):14308–13. 10.1073/pnas.1306290110 PMC3761611 23940335

[8] Tackney JC, Potter BA, Raff J, Powers M, Watkins WS, Warner D, et al Two contemporaneous mitogenomes from terminal Pleistocene burials in eastern Beringia. Proceedings of the National Academy of Sciences. 2015;112(45):13833–8. 10.1073/pnas.1511903112 PMC4653186 26504230

[9] Raghavan M, Steinrücken M, Harris K, Schiffels S, Rasmussen S, DeGiorgio M, et al Genomic evidence for the Pleistocene and recent population history of Native Americans. Science. 2015;349(6250). 10.1126/science.aab3884 PMC4733658 26198033

[10] Llamas B, Fehren-Schmitz L, Valverde G, Soubrier J, Mallick S, Rohland N, et al Ancient mitochondrial DNA provides high-resolution time scale of the peopling of the Americas. Science advances. 2016;2(4):e1501385 10.1126/sciadv.1501385 27051878 PMC4820370

[11] Scott GR, Schmitz K, Heim KN, Paul KS, Schomberg R, Pilloud MA. Sinodonty, Sundadonty, and the Beringian Standstill model: Issues of timing and migrations into the New World. Quaternary International. 2016. Available online 27 May 2016, ISSN 1040-6182,

[12] Hoffecker JF, Elias SA, O'Rourke DH, Scott GR, Bigelow NH. Beringia and the global dispersal of modern humans. Evolutionary Anthropology. 2016;25(2):64–78. 10.1002/evan.21478 27061035

[13] Pitulko VV, Tikhonov AN, Pavlova EY, Nikolskiy PA, Kuper KE, Polozov RN. Early human presence in the Arctic: Evidence from 45,000-year-old mammoth remains. Science. 2016;351(6270):260–3. 10.1126/science.aad0554 26816376

[14] Pitulko VV, Nikolsky PA, Girya EY, Basilyan AE, Tumskoy VE, Koulakov SA, et al The Yana RHS site: humans in the Arctic before the Last Glacial Maximum. Science. 2004;303(5654):52–6. 10.1126/science.1085219 14704419

[15] Nikolskiy P, Pitulko V. Evidence from the Yana Palaeolithic site, Arctic Siberia, yields clues to the riddle of mammoth hunting. Journal of Archaeological Science. 2013;40:4189–97.

[16] Pitulko VV. The Berelekh quest: A review of forty years of research in the mammoth graveyard in northeast Siberia. Geoarchaeology Review Paper. 2011;26(1):5–32.

[17] Pitulko VV, Basilyan AE, Pavlova EY. The Berelekh Mammoth “Graveyard”: New Chronological and Stratigraphical Data from the 2009 Field Season. Geoarchaeology. 2014;29(4):277–99.

[18] Goebel T, Slobodin SB, Waters MR. New dates from Ushki-1, Kamchatka, confirm 13,000 calBP age for earliest Paleolithic occupation. Journal of Archaeological Science. 2010;37(10):2640–9.

[19] Yesner DR. Human dispersal into interior Alaska: antecedent conditions, mode of colonization, and adaptations. Quaternary Science Reviews. 2001;20(1–3):315–27.

[20] Holmes CE. The Beringian and transitional periods in Alaska: technology of the East Beringian Tradition as viewed from Swan Point In: Goebel T, Buvit I, editors. From the Yenisei to the Yukon: Interpreting lithic assemblage variability in Late Pleistocene/Early Holocene Beringia: Center for the Study of the First Americans, Texas A&M University Press, College Station, Texas; 2011 p. 179–91.

[21] Potter BA, Holmes CE, Yesner DR. Technology and Economy Among the Earliest Prehistoric Foragers in Interior Eastern Beringia In: Graf KE, Ketron CV, Waters MR, editors. Paleoamerican Odyssey: Texas A&M University Press; 2013 p. 81–103.

[22] Easton NA, MacKay GR, Young PB, Schnurr P, Yesner DR. Chindadn in Canada—emergent evidence of the Pleistocene transition of Southeast Beringia as revealed by the Little John Site (KdVo-6), Yukon Territory, Canada In: Goebel T, Buvit I, editors. From the Yenisei to the Yukon: Interpreting Lithic Assemblage Variability in Late Pleistocene/Early Holocene Beringia: Center for the Study of the First Americans, Texas A&M University Press, College Station, Texas; 2011 p. 289–307.

[23] Cinq-Mars J. Bluefish Cave I: a late Pleistocene eastern Beringian cave deposit in the northern Yukon. Canadian Journal of Archaeology. 1979;3:1–32.

[24] Cinq-Mars J. La place des grottes du Poisson-Bleu dans le Préhistoire béringienne. Revista de Arqueología Americana. 1990;1:9–32.

[25] Cinq-Mars J, Morlan RE. Bluefish Caves and Old Crow Basin: A new rapport In: Bonnichsen R, Turnmire KL, editors. Ice Age Peoples of North America Environments, Origins, and Adaptations of the First Americans: Center for the Study of the First Americans. Oregon State University Press; 1999 p. 200–12.

[26] Morlan RE, Cinq-Mars J. Ancient Beringians: human occupation in the Late Pleistocene of Alaska and the Yukon Territory In: Hopkins DM, Matthews JV, Schweger CE, Young SB, editors. Paleoecology of Beringia New York: Academic Press; 1982 p. 353–81.

[27] Harington CR, Cinq-Mars J. Bluefish Caves—Fauna and context. Beringian Research Notes. 2008;19:8 p.

[28] Canadian Museum of History, Archives, Jacques Cinq-Mars fonds, « Artifact Catalogues: P.B.I–MgVo-1, P.B.II–MgVo-2 », boxes 2 and 3.

[29] Morlan RE. Current perspectives on the Pleistocene archaeology of eastern Beringia. Quaternary Research. 2003;60(1):123–32.

[30] Gomez-Coutouly YA. Industries lithiques à composante lamellaire par pression du Nord Pacifique de la fin du Pléistocène au début de l'Holocène: de la diffusion d'une technique en Extrême-Orient au peuplement initial du Nouveau Monde. Thèse de Doctorat, Université Paris Ouest, Nanterre La Défense; 2011.

[31] Ritchie JC, Cinq-Mars J, Cwynar LC. L’environnement tardiglaciaire du Yukon septentrional, Canada Géographie physique et Quaternaire. 1982;36(1–2):241–50.

[32] Zazula GD, Schweger CE, Beaudoin AB, McCourt GH. Macrofossil and pollen evidence for full-glacial steppe within an ecological mosaic along the Bluefish River, eastern Beringia. Quaternary International. 2006;142–143:2–19.

[33] Guthrie RD. Origin and causes of the mammoth steppe: a story of cloud cover, woolly mammal tooth pits, buckles, and inside-out Beringia. Quaternary Science Reviews. 2001;20(1–3):549–74.

[34] Harington CR. Pleistocene vertebrates of the Yukon Territory. Quaternary Science Reviews. 2011;30:2341–54.

[35] Burke A, Cinq-Mars J. Paleoethological Reconstruction and Taphonomy of *Equus lambei* from the Bluefish Caves, Yukon Territory, Canada. Arctic. 1998;51(2):105–15.

[36] Dixon EJ. Bones, boats & bison: archeology and the first colonization of western North America. Albuquerque: University of New Mexico Press; 1999. 322 p.

[37] Hoffecker JF, Elias SA. The human ecology of Beringia: Columbia University Press; 2007. 290 p.

[38] Goebel T, Waters MR, O'Rourke DH. The late Pleistocene dispersal of modern humans in the Americas. Science. 2008;319(5869):1497–502. 10.1126/science.1153569 18339930

[39] Bourgeon L. Bluefish Cave II (Yukon Territory, Canada): taphonomic study of a bone assemblage. PaleoAmerica. 2015;1(1):105–8.

[40] Shipman P, Rose JJ. Bone tools: an experimental approach Scanning electron microscopy in archaeology. Oxford, England: BAR International Series 452; 1988 p. 303–35.

[41] Fisher JW. Bone surface modifications in zooarchaeology. Journal of Archaeological Method and Theory. 1995;2(1):7–68.

[42] Morlan RE. Taphonomy and archaeology in the Upper Pleistocene of the Northern Yukon Territory: A glimpse of the peopling of the New World. Ottawa: National Museum of Man Mercury Series Archaeological Survey of Canada; 1980. 398 p.

[43] Olsen SL, Shipman P. Surface modification on bone: trampling versus butchery. Journal of Archaeological Science. 1988;15(5):535–53.

[44] Domínguez-Rodrigo M, De Juana S, Galán A, Rodríguez M. A new protocol to differentiate trampling marks from butchery cut marks. Journal of Archaeological Science. 2009;36(12):2643–54.

[45] Hockett B, Jenkins DL. Identifying stone tool cut marks and the pre-Clovis occupation of the Paisley Caves. American Antiquity. 2013;78(4):762–78.

[46] Monnier GF, Bischoff E. Size matters. An evaluation of descriptive and metric criteria for identifying cut marks made by unmodified rocks during butchery. Journal of Archaeological Science. 2014;50:305–17.

[47] Capaldo SD, Blumenschine RJ. A quantitative diagnosis of notches made by hammerstone percussion and carnivore gnawing on bovid long bones. American Antiquity. 1994;59(4):724–48.

[48] Binford LR. Bones. Ancient men and modern myths. New York: Academic Press; 1981. 320 p.

[49] Haynes G. Evidence of carnivore gnawing on Pleistocene and Recent mammalian bones. Paleobiology. 1980;6(3):341–51.

[50] Haynes G. A guide for differentiating mammalian carnivore taxa responsible for gnaw damage to herbivore limb bones. Paleobiology. 1983;9(2):164–72.

[51] Domínguez-Rodrigo M, Barba R. New estimates of tooth mark and percussion mark frequencies at the FLK Zinj site: the carnivore-hominid-carnivore hypothesis falsified. Journal of Human Evolution. 2006;50(2):170–94. 10.1016/j.jhevol.2005.09.005 16413934

[52] Duches R, Nannini N, Romandini M, Boschin F, Crezzini J, Peresani M. Identification of Late Epigravettian hunting injuries: Descriptive and 3D analysis of experimental projectile impact marks on bone. Journal of Archaeological Science. 2016;66:88–102.

[53] Behrensmeyer AK. Taphonomic and ecologic information from bone weathering. Paleobiology. 1978;4(2):150–62.

[54] Todisco D, Monchot H. Bone weathering in a periglacial environment: the Tayara site (KbFk-7), Qikirtaq Island, Nunavik (Canada). Arctic. 2008;61(1):87–101.

[55] Guadelli JL. La gélifraction des restes fauniques. Expérimentation et transfert au fossile. Annales de Paléontologie 2008;94:121–65.

[56] Lyman RL. Archaeofaunas and butchery studies: a taphonomic perspective. Advances in archaeological method and theory. 1987;10:249–337.

[57] Walker PL, Long JC. An experimental study of the morphological characteristics of tool marks. American antiquity. 1977;42(4):605–16.

[58] Boschin F, Crezzini J. Morphometrical analysis on cut marks using a 3D digital microscope. International Journal of Osteoarchaeology. 2012;22(5):549–62.

[59] Bello SM, Soligo C. A new method for the quantitative analysis of cutmark micromorphology. Journal of Archaeological Science. 2008;35(6):1542–52.

[60] Wheat J. The Jurgens Site. Plains Anthropologist. 1979;24(84):1–153.

[61] Abe Y. Hunting and butchery patterns of the Evenki in Northern Transbaikalia, Russia. Ph.D. Thesis, New-York: Stony Brook University; 2005.

[62] Costamagno S, David F. Comparaison des pratiques bouchères et culinaires de différents groupes sibériens vivant de la renniculture. Archaeofauna. 2009;18:9–25.

[63] Merritt SR. Factors affecting Early Stone Age cut mark cross-sectional size: implications from actualistic butchery trials. Journal of Archaeological Science. 2012;39(9):2984–94.

[64] Braun DR, Pante M, Archer W. Cut marks on bone surfaces: influences on variation in the form of traces of ancient behaviour. Interface focus. 2016;6(3):20160006 10.1098/rsfs.2016.0006 27274806 PMC4843629

[65] Brown TA, Nelson DE, Vogel JS, Southon JR. Improved collagen extraction by modified Longin method. Radiocarbon. 1988;30(2):171–7.

[66] Brock F, Higham T, Ditchfield P, Bronk Ramsey C. Current pretreatment methods for AMS radiocarbon dating at the Oxford Radiocarbon Accelerator Unit (ORAU). Radiocarbon. 2010;52(1):103–12.

[67] Bronk Ramsey C. Development of the radiocarbon calibration program. Radiocarbon. 2001;43(2A):355–64.

[68] Reimer PJ, Bard E, Bayliss A, Beck JW, Blackwell PG, Bronk Ramsey C, et al IntCal13 and Marine13 radiocarbon age calibration curves 0–50,000 years cal BP. Radiocarbon. 2013;55(4):1869–87.

[69] McCuaig-Balkwill D, Cinq-Mars J. Migratory birds from Bluefish Caves, Eastern Beringia. 8th International Congress of the International Council for Archaeozoology Final Program and Abstracts; August 23–29, Victoria, B.C.1998. p. 9.

[70] Stuiver M, Polach HA. Discussion; reporting of C-14 data. Radiocarbon. 1977;19(3):355–63.

[71] Bronk Ramsey C, Higham T, Leach P. Towards high-precision AMS: progress and limitations. Radiocarbon. 2004;46(1):17–24.

[72] Coplen TB. Reporting of stable hydrogen, carbon, and oxygen isotopic abundances (technical report). Pure and Applied Chemistry. 1994;66(2):273–6.

[73] Endacott NA. The zooarchaeology of Lime Hills Cave: paleoecological and taphonomic insights. Ph.D. Thesis, Washington State University; 2008.

[74] Sattler RA. Large mammals in Lower Rampart Cave 1, Alaska: interspecific utilization of an eastern Beringian cave. Geoarchaeology. 1997;12(6):657–88.

[75] Potter BA. Models of faunal processing and economy in Early Holocene interior Alaska. Environmental Archaeology. 2007;12(1):3–23.

[76] Vinson DM. Taphonomic analysis of faunal remains from Trail Creek caves, Seward Peninsula, Alaska. M.A. Thesis, University of Alaska Fairbanks; 1993.

[77] Mandryk CAS, Josenhans H, Fedje DW, Mathewes RW. Late Quaternary paleoenvironments of Northwestern North America: implications for inland versus coastal migration routes. Quaternary Science Reviews. 2001;20(1–3):301–14.

[78] Perdersen MW, Ruter A, Schweger CE, Friebe H, Staff RA, Kjeldsen KK, et al Postglacial viability and colonization in North America's ice-free corridor. Nature. 2016;537(7618). 10.1038/nature19085 27509852

[79] Heintzman PD, Froese D, Ives JW, Soares AER, Zazula GD, Letts B, et al Bison phylogeography constrains dispersal and viability of the Ice Free Corridor in western Canada. Proceedings of the National Academy of Sciences. 2016;113(29):8057–63. 10.1073/pnas.1601077113 PMC4961175 27274051

[80] Guthrie RD. New carbon dates link climatic change with human colonization and Pleistocene extinctions. Nature. 2006;441(7090):207–9. 10.1038/nature04604 16688174

