# Supplementary material for: Earliest Human Presence in North America Dated to the Last Glacial Maximum: New Radiocarbon Dates from Bluefish Caves, Canada
Source: PLoS One. 2017 Jan 6;12(1):e0169486. doi: 10.1371/journal.pone.0169486 (PMC5218561; doi:10.1371/journal.pone.0169486)

**S1 Figs. Cut-marked bone specimens from Bluefish Cave I (A-J) and Cave II (K-M).** The faunal collections from Bluefish Caves are curated at the Canadian Museum of History (Gatineau, QC). Taphonomic analyses were conducted in the Ecomorphology and Paleoanthropology laboratory (U. de Montréal) and high resolution digital images were taken using the Olympus DSX-100 microscope.


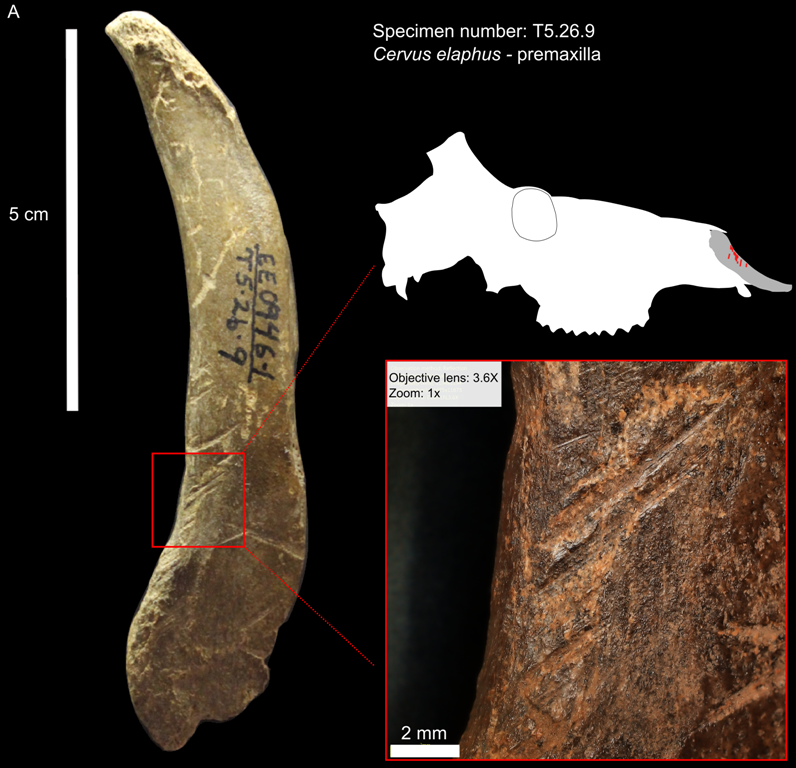

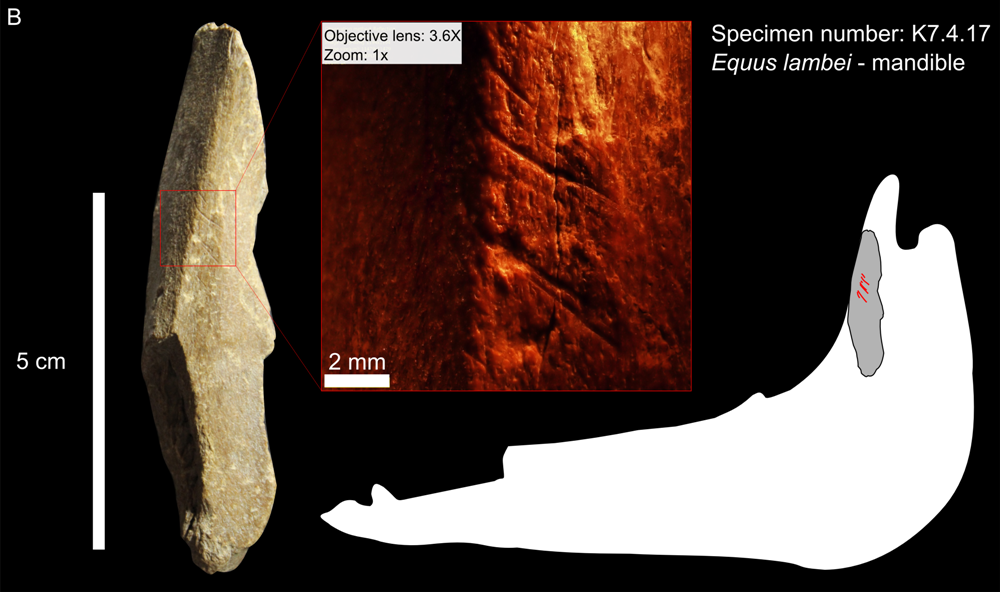

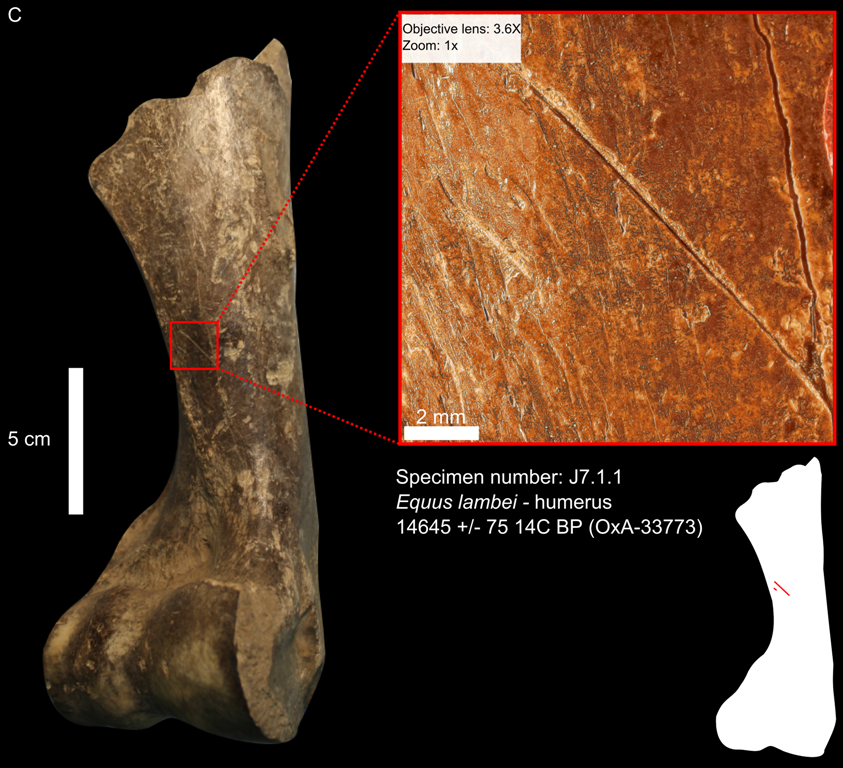

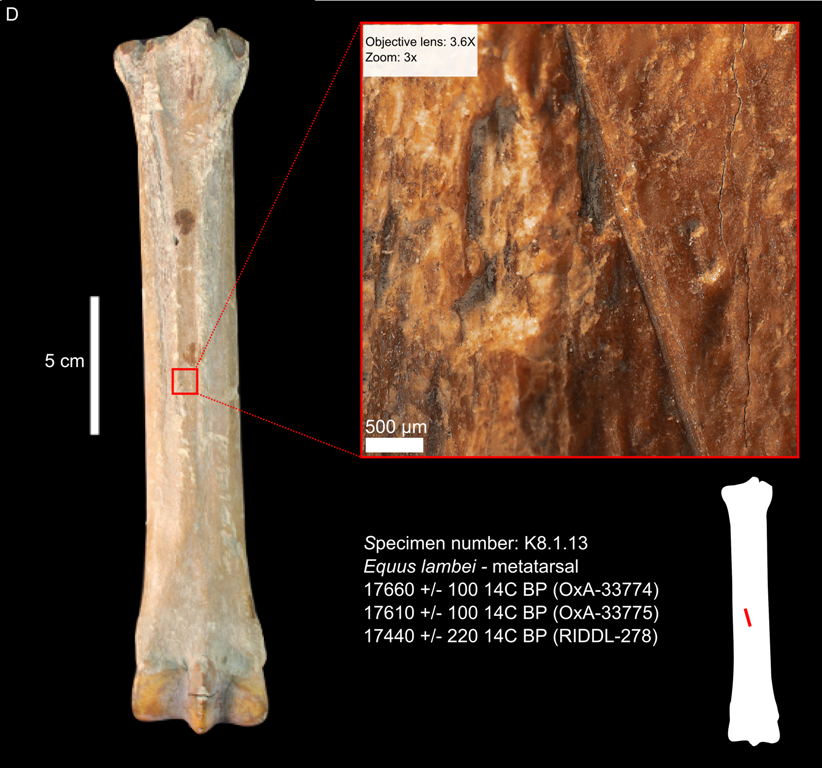

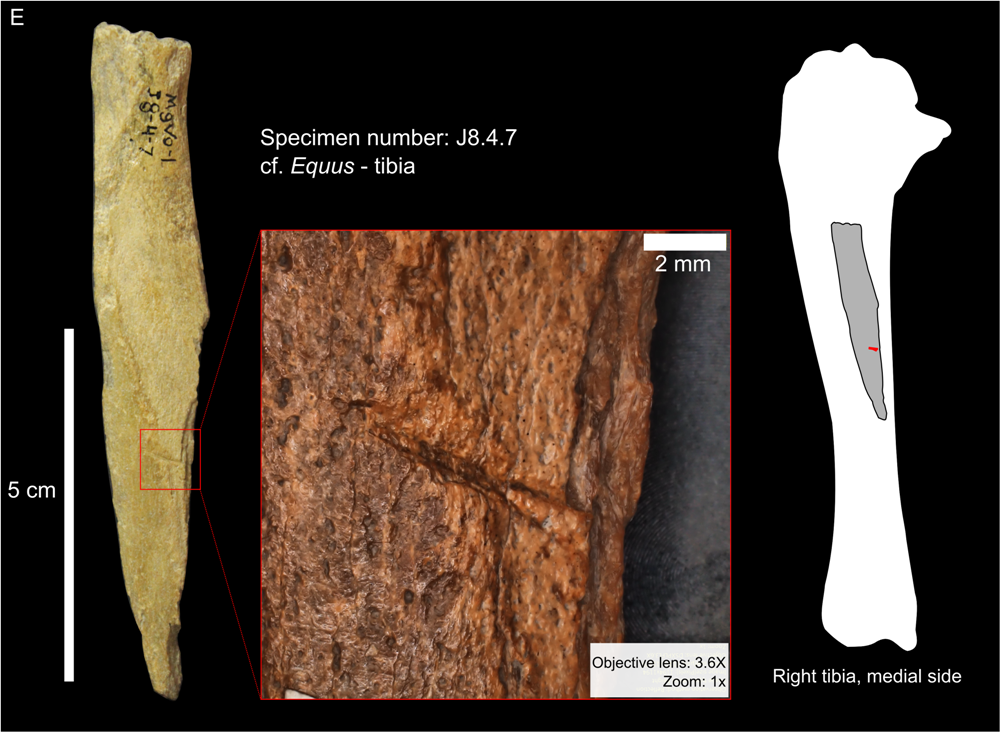

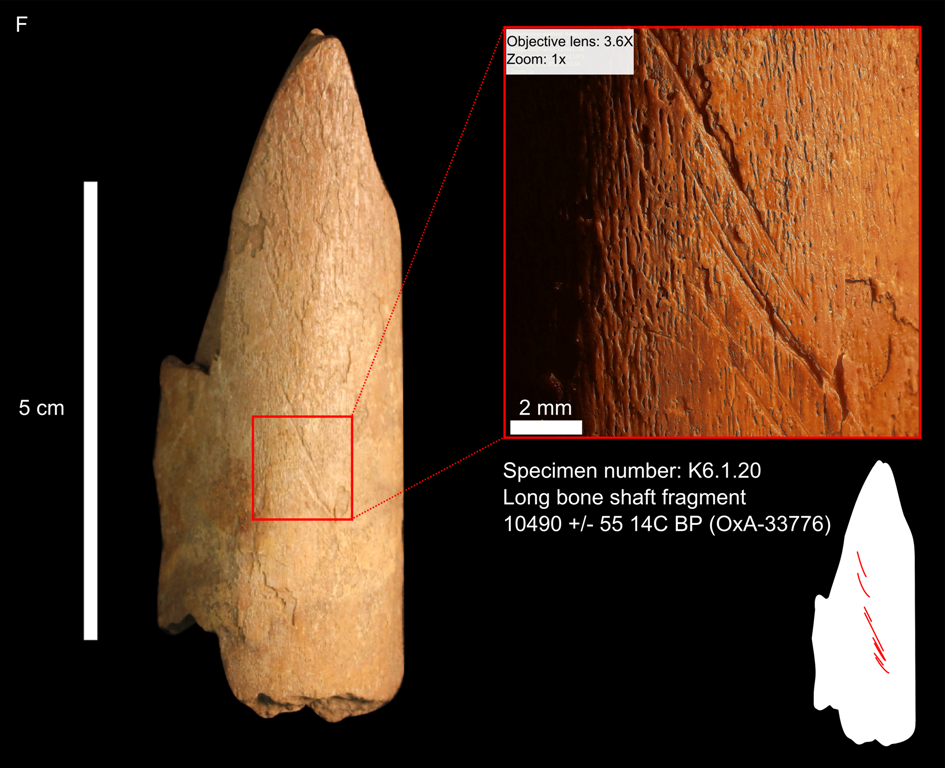

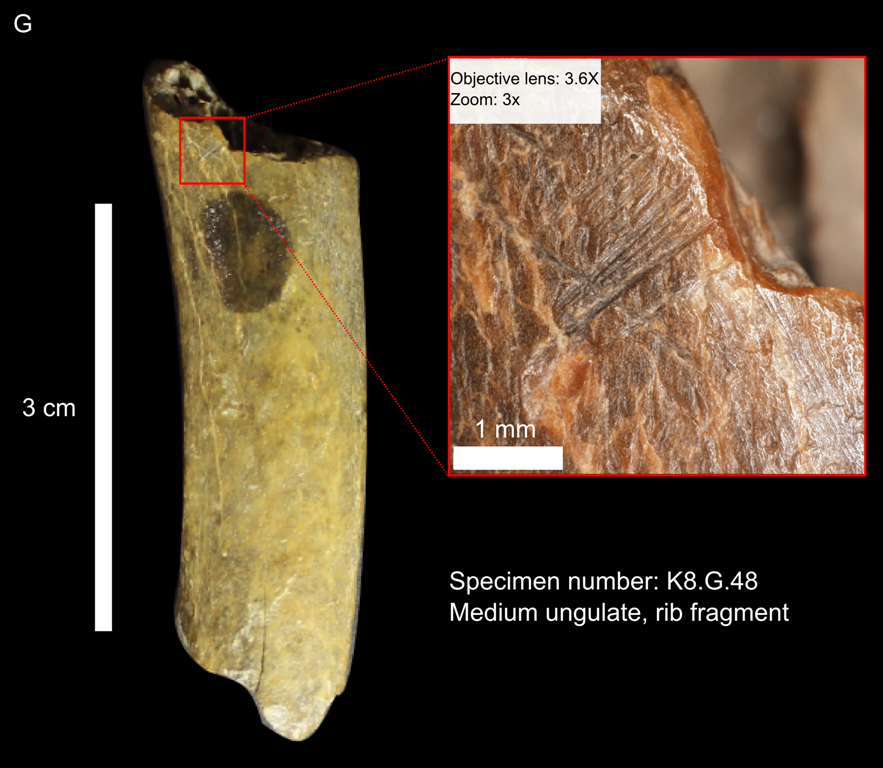

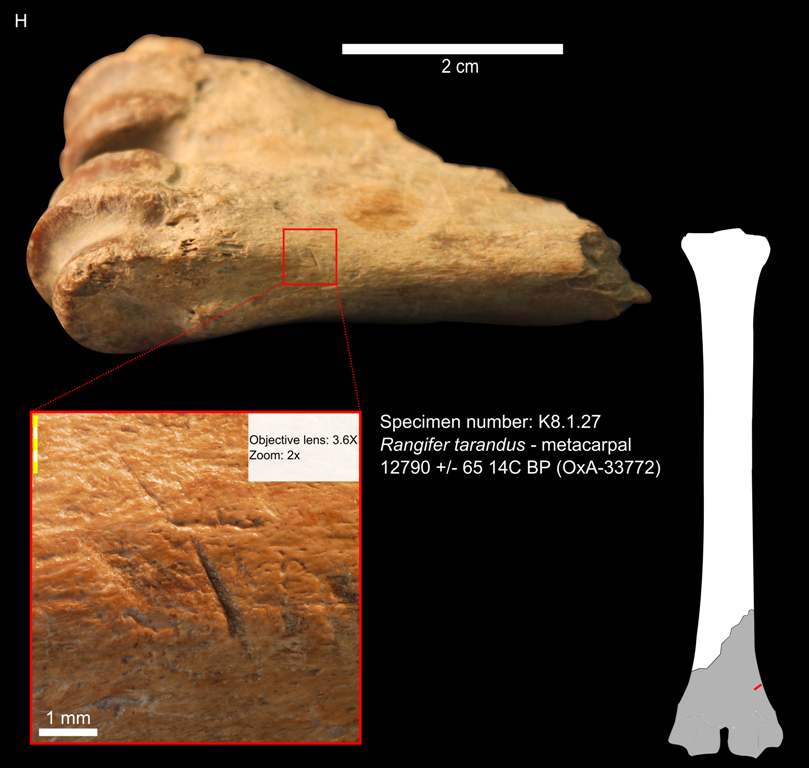

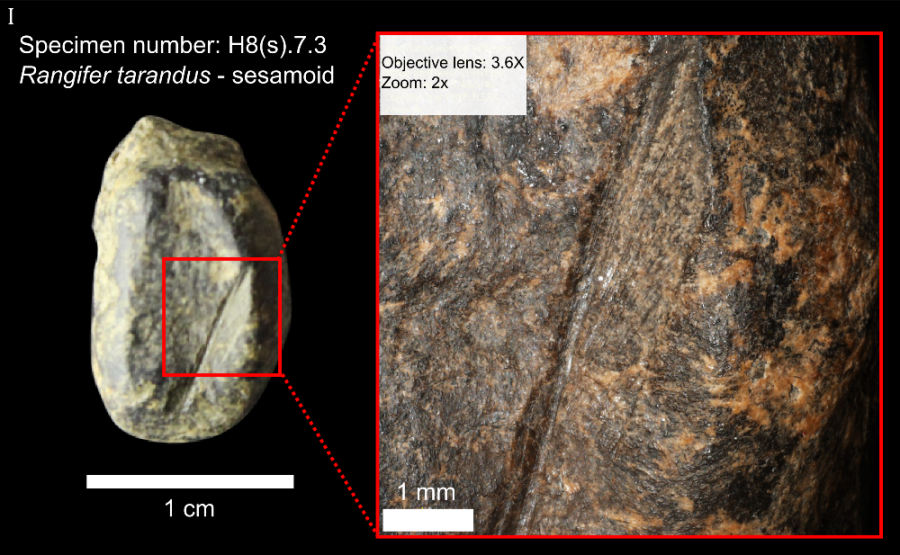

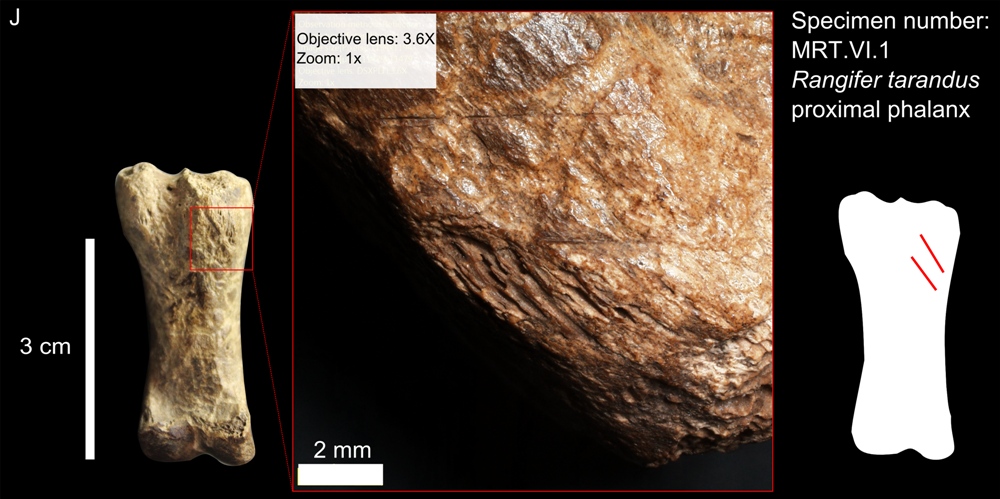

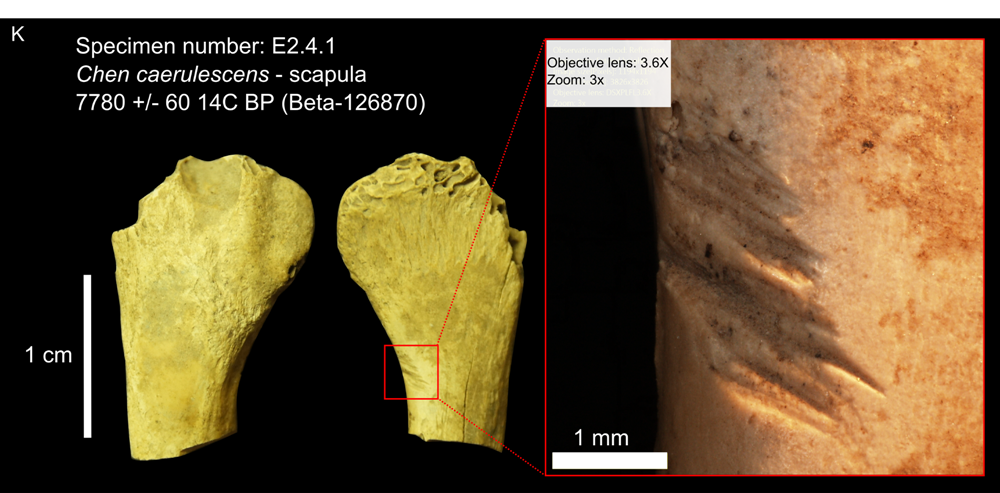

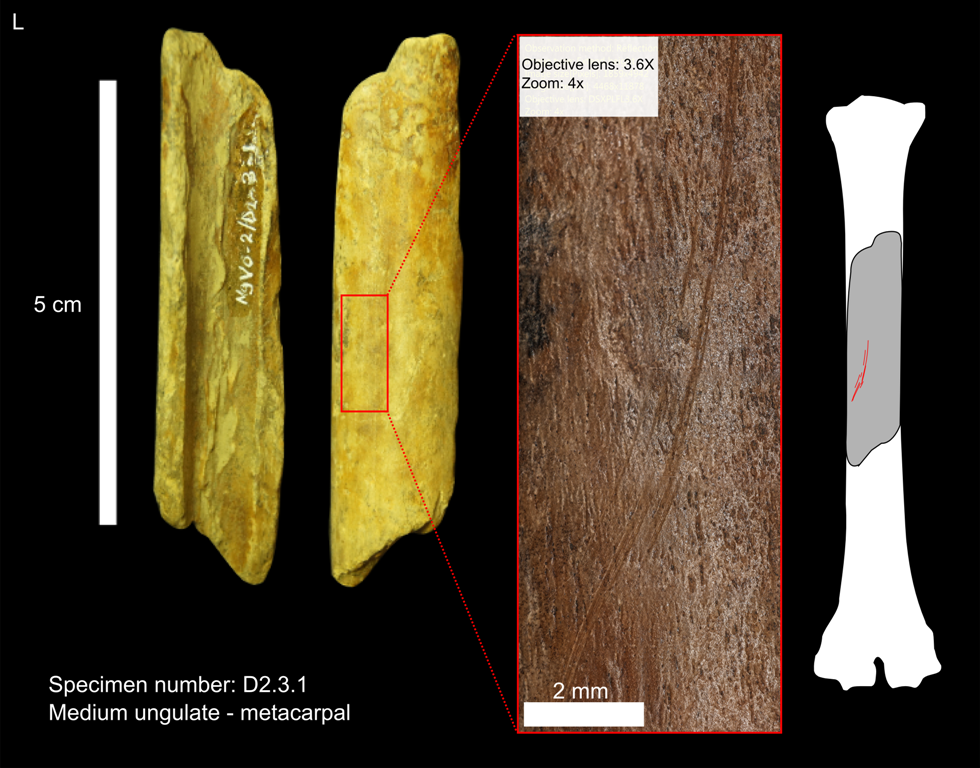

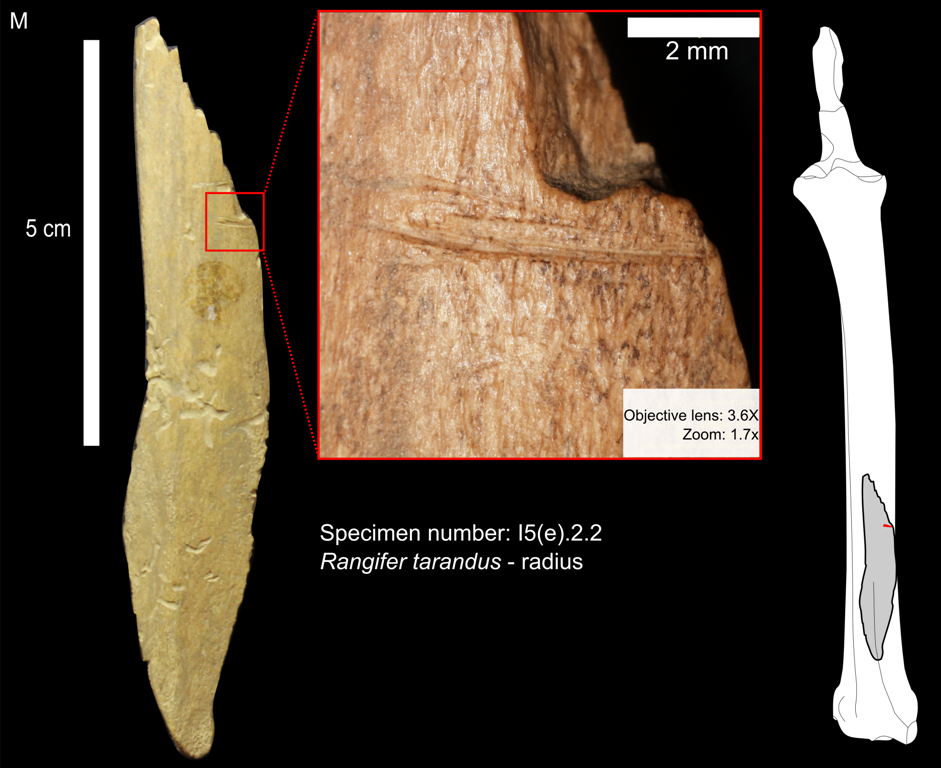

Supplement: S1 Fig — The faunal collections from Bluefish Caves are curated at the Canadian Museum of History (Gatineau, QC). Taphonomic analyses were conducted in the Ecomorphology and Paleoanthropology laboratory (U. de Montréal) and high resolution digital images were taken using the Olympus DSX-100 microscope. (DOCX) [file pone.0169486.s001.docx]
