# Supplementary material for: Earliest Human Presence in North America Dated to the Last Glacial Maximum: New Radiocarbon Dates from Bluefish Caves, Canada
Source: PLoS One. 2017 Jan 6;12(1):e0169486. doi: 10.1371/journal.pone.0169486 (PMC5218561; doi:10.1371/journal.pone.0169486)
Supplement: S1 Graph — Our taphonomic analysis began with the faunal material of Bluefish Cave II and was applied to each specimen greater than 20 mm in length (N = 5980) [see also ref. 35]. Because of time constraints, the same methodology was applied to the bone specimens of Cave I measuring more than 30 mm in length (N = 5425). (DOCX) [file pone.0169486.s002.docx]

**S1 Graph. Impact of the natural taphonomic processes affecting the bone assemblages.** Our taphonomic analysis began with the faunal material of Cave II and was applied to each specimen greater than 20 mm in length (N = 5980) [see also ref. 35]. Because of time constraints, the same methodology was applied to the bone specimens of Cave I measuring more than 30 mm in length (N = 5425).
