# Supplementary material for: Earliest Human Presence in North America Dated to the Last Glacial Maximum: New Radiocarbon Dates from Bluefish Caves, Canada
Source: PLoS One. 2017 Jan 6;12(1):e0169486. doi: 10.1371/journal.pone.0169486 (PMC5218561; doi:10.1371/journal.pone.0169486)
Supplement: S2 Graph — Measurements obtained on fourteen cut-marked bone specimens from Bluefish Caves I and II (see S2 Table) are compared to the ranges reported for experimental and archaeological data: (1) carnivore tooth marks [48], (2) experimental steel blade, (3) archaeological data from an Italian site dated to the Iron Age (i.e. Trebbio), (4) experimental flint flakes, (5) experimental retouched tool, (6) archaeological data from an Italian site dated to the Paleolithic (i.e. Paglicci) [52], (7) cut marks from Bluefish Caves. Our graphs show that the depth and opening angle we measured are in the range reported by comparative studies [48, 52]. The breadth ratio (the ratio between the breadth at the top and the breadth at the bottom of the cut mark) is a better criterion for distinguishing between cut marks made with stone tools and modifications produced by other effectors [48, 52]. Here, we show that the relatively high breadth ratios that we obtained from Bluefish Caves are comparable to the measures obtained on cut marks produced by flint flakes and retouched tool and indicate the presence of “V-shaped” grooves [52]. (DOCX) [file pone.0169486.s003.docx]

**S2 Graph. Morphometrical analysis.** Measurements obtained on fourteen cut-marked bone specimens from Bluefish Caves I and II (see S2 Table) are compared to the ranges reported for experimental and archaeological data: (1) carnivore tooth marks [48], (2) experimental steel blade, (3) archaeological data from an Italian site dated to the Iron Age (i.e. Trebbio), (4) experimental flint flakes, (5) experimental retouched tool, (6) archaeological data from an Italian site dated to the Paleolithic (i.e. Paglicci) [52], (7) cut marks from Bluefish Caves. Our graphs show that the depth and opening angle we measured are in the range reported by comparative studies [48, 52]. The breadth ratio (the ratio between the breadth at the top and the breadth at the bottom of the cut mark) is a better criterion for distinguishing between cut marks made with stone tools and modifications produced by other effectors [48, 52]. Here, we show that the relatively high breadth ratios that we obtained from Bluefish Caves are comparable to the measures obtained on cut marks produced by flint flakes and retouched tool and indicate the presence of “V-shaped” grooves [52].
