# Supplementary material for: Earliest Human Presence in North America Dated to the Last Glacial Maximum: New Radiocarbon Dates from Bluefish Caves, Canada
Source: PLoS One. 2017 Jan 6;12(1):e0169486. doi: 10.1371/journal.pone.0169486 (PMC5218561; doi:10.1371/journal.pone.0169486)
Supplement: S1 Table — Morphological features described in the article were noted for each cut mark observed on the bone specimens from Bluefish Caves I and II. (DOCX) [file pone.0169486.s004.docx]

**S1 Table. Morphological analysis.** Morphological features described in the article were noted for each cut mark observed on the bone specimens from Bluefish Caves I and II.

*Specimens dated in this study.

| Cave | Specimen number | Trajectory: straight (T), curved (C), sinuous (S) | Orientation: longitudinal (L), transverse (T), oblique (O) | Number: single (S), multiple (M) and parallel (P) | Overlapping striae: yes (Y), no (N) | Shoulder effect: yes (Y), no (N) | Shoulder flaking: yes (Y), no (N) | Internal microstriations: yes (Y), no (N) |
| --- | --- | --- | --- | --- | --- | --- | --- | --- |
| 1 | T5.26.9 | T | O | MP | N | N | N | Y |
|  | K7.4.17 | C | O | MP | N | N | N | N |
|  | J7.1.1* | T | O | MP | N | Y | Y | Y |
|  | K8.1.13* | T | O | S | N | N | N | Y |
|  | J8.4.7 | T | T | MP | Y | N | N | N |
|  | K6.1.20* | C | O | MP | N | ? | ? | Y |
|  | K8.G.48 | T | O | MP | Y | N | N | Y |
|  | K8.1.27* | T | O | S | N | Y | N | N |
|  | H8(s).7.3 | T | L | S | N | N | N | Y |
|  | MRT.VI.1 | T | O | MP | N | ? | ? | ? |
| 2 | E2.4.1 | T | O | MP | Y | N | N | N |
|  | I5(e).6.5* | T | L | MP | N | Y | Y | Y |
|  | D2.3.1 | C | L | MP | N | Y | N | Y |
|  | J7.8.17* | T | O | MP | N | Y | Y | Y |
|  | I5(e).2.2 | T | T | S | N | Y | N | N |
