# Supplementary material for: Earliest Human Presence in North America Dated to the Last Glacial Maximum: New Radiocarbon Dates from Bluefish Caves, Canada
Source: PLoS One. 2017 Jan 6;12(1):e0169486. doi: 10.1371/journal.pone.0169486 (PMC5218561; doi:10.1371/journal.pone.0169486)
Supplement: S2 Table — The depth, breadth and opening angle were measured on fourteen bone specimens from Bluefish Caves I and II bearing cultural modifications confidently attributable to human activities. Measurements were obtained on the median cross-section of cut marks [as described in ref. 52], using the Olympus DSX-100 microscope (16x optical zoom; objective lens: 3.6X). When the bone surface was too altered or when multiple incisions were present, a second measure was taken. (DOCX) [file pone.0169486.s005.docx]

**S2** **Table . Morphometrical analysis.** The depth, breadth and opening angle were measured on fourteen bone specimens from Bluefish Caves I and II bearing cultural modifications confidently attributable to human activities. Measurements were obtained on the median cross-section of cut marks [as described in ref. 52], using the Olympus DSX-100 microscope (16x optical zoom; objective lens: 3.6X). When the bone surface was too altered or when multiple incisions were present, a second measure was taken.

*Specimens dated in this study.

| **Cave** | **Specimen number** | **Depth**  **(µm)** | **Breadth at the bottom**  **(µm)** | **Breadth at the top**  **(µm)** | **Breadth ratio (top/bottom)**  **(µm)** | **Opening angle**  **(°)** |
| --- | --- | --- | --- | --- | --- | --- |
| 1 | T5.26.9 (1) | 51,7 | 29,9 | 702,5 | 23,5 | 150,4 |
|  | T5.26.9 (2) | 41,7 | 32,4 | 428,5 | 13,2 | 146,8 |
|  | K7.4.17 | 40,5 | 17,4 | 371,2 | 21,3 | 140,9 |
|  | J7.1.1* (1) | 72,0 | 24,9 | 637,7 | 25,6 | 141,7 |
|  | J7.1.1* (2) | 106,9 | 14,9 | 423,5 | 28,4 | 106,6 |
|  | K8.1.13* (1) | 78,7 | 29,9 | 627,8 | 21,0 | 141,3 |
|  | K8.1.13* (2) | 73,9 | 14,9 | 637,7 | 42,7 | 140,1 |
|  | J8.4.7 (1) | 202,3 | 74,7 | 1310,3 | 17,5 | 130,0 |
|  | J8.4.7 (2) | 234,7 | 84,7 | 1629,2 | 19,2 | 139,3 |
|  | K6.1.20* | 94,2 | 29,9 | 886,8 | 29,7 | 138,6 |
|  | K8.G.48 (1) | 18,3 | 8,5 | 144,1 | 17,0 | 142,1 |
|  | K8.G.48 (2) | 42,9 | 10,5 | 352,7 | 33,7 | 149,0 |
|  | K8.1.27* | 84,1 | 14,9 | 612,8 | 41,0 | 130,5 |
|  | H8(s).7.3 | 93,0 | 18,3 | 554,7 | 30,3 | 128,7 |
|  | MRT.VI.1 | 124,6 | 34,9 | 924,2 | 26,5 | 136,2 |
| 2 | E2.4.1 (1) | 98,3 | 54,8 | 876,9 | 16,0 | 144,2 |
|  | E2.4.1 (2) | 139,5 | 22,4 | 677,6 | 30,2 | 130,8 |
|  | I5(e).6.5* (1) | 100,4 | 59,8 | 906,8 | 15,2 | 139,3 |
|  | I5(e).6.5* (2) | 89,8 | 29,9 | 722,4 | 24,2 | 138,5 |
|  | J7.8.17* (1) | 106,0 | 114,6 | 921,7 | 8,0 | 139,2 |
|  | J7.8.17* (2) | 75,0 | 19,9 | 697,5 | 35,0 | 149,0 |
|  | I5(e).2.2 (1) | 122,1 | 24,9 | 789,7 | 31,7 | 142,8 |
|  | I5(e).2.2 (2) | 112,9 | 53,1 | 782,2 | 14,7 | 130,0 |
