# Supplementary material for: Earliest Human Presence in North America Dated to the Last Glacial Maximum: New Radiocarbon Dates from Bluefish Caves, Canada
Source: PLoS One. 2017 Jan 6;12(1):e0169486. doi: 10.1371/journal.pone.0169486 (PMC5218561; doi:10.1371/journal.pone.0169486)
Supplement: S3 Table — Descriptions of the cut marks observed on the bone specimens from Bluefish Caves I and II and comparisons with ethnographic data. (DOCX) [file pone.0169486.s006.docx]

**S3 Table. Ethnographic comparisons.** Descriptions of the cut marks observed on the bone specimens from Bluefish Caves I and II and comparisons with ethnographic data.

*Specimens dated in this study.

| **Bluefish Cave I** | | | |
| --- | --- | --- | --- |
| **Element and spec. number** | **Taxon** | **Observations** | **Ethnographic comparisons** |
| Premaxilla (complete bone) # T5.26.9 | *Cervus canadensis* | Multiple parallel cut marks on the lateral face = removal of the skin around the muzzle | Binford 1981:107: "[…] cut marks if the head is in fact skinned out […] around the mouth, particularly in the "chin" area of the mandible." See also Abe 2005:468, Figure 7.31 for butchery marks on deer skull. |
| Mandible (fragment) # K7.4.17 | *Equus lambei* | Eight fine and shallow cut marks transversal to the bone, on the anterior border of the ramus = dismemberment of the mandible | Binford 1981:109: "[…] the masseter muscle was cleaned off the mandible as well as stripped off its insertion points on the skull. This resulted in cut marks around the edges of the masseteric fossa […]". |
| Humerus* (shaft + distal end) # J7.1.1 | *Equus lambei* | Two straight, deep and oblique cut marks on the anterior face of the shaft = filleting | Binford 1981:141, Tabl. 4.04 : "Oblique short "chevron" marks clustered on neck of distal end on the anterior face". See also Wheat 1979:63, Figure 31 for butchering marks produced on a bison humerus. |
| Metatarsal* (complete bone) # K8.1.13 | *Equus lambei* | One long and deep cut mark on the posterior face of the canon bone = possible stripping of tendons | Binford 1981:95: "The sheath of tendons running in the ventral channel of the canon bone is stripped up to the proximal end of the bone. Cuts are then made between the sheath and the bone […]". See also Wheat 1979:66, Figure 32 for butchering marks produced on a bison metapodial. |
| Tibia (? shaft fragment) # J8.4.7 | cf. *Equus* | Two short, deep and transverse cut marks overlapping on a probable medial face of a tibia shaft fragment = possible filleting | Binford 1981:129-130 : " […] short cut marks frequently made obliquely to the longitudinal aspect of the bone and concentrated on both the anterior and posterior faces. […] clustered where the shape of the bone is irregular and where they are numerous muscle insertions." |
| Long bone shaft fragment* # K6.1.20 | Large ungulate (cf. *Cervus*) | Eight traces diagonally oriented to the long axis of the shaft = filleting | Binford 1981:129: " […] short cut marks frequently made obliquely to the longitudinal aspect of the bone and concentrated on both the anterior and posterior faces." |
| Rib (fragment) # K8.G.48 | cf. *Rangifer* | Small area of bidirectional short marks on the lateral face of the rib = filleting | Wheat 1979:68: "Areas of light diagonal scratches were found on the lateral surfaces […]" |
| Metacarpal* (distal end) # K8.1.27 | *Rangifer tarandus* | One short and oblique incision on the lateral or medial side of the distal end = filleting | Binford 1981:140, Tabl. 4.04: "Short "chevron" marks obliquely clustered on lateral and medial crests, anterior aspect". See also Binford 1981:132, Figure 4.38 shows butchering marks produced on a distal metatarsal of caribou during filleting. |
| Sesamoïd # H8(s).7.3 | *Rangifer tarandus* | One large and deep cut, longitudinally oriented = skinning | Costamagno and David 2009:15-16: "Sur les extrémités des pattes, des stries longitudinales présentes sur la face postérieure des métapodes, des premières et deuxièmes phalanges sont consécutives à la découpe de la peau". |
| Proximal phalanx # MRT.VI.1 | *Rangifer tarandus* | Two cut marks obliquely oriented on the ventral surface near the proximal end = skinning | Costamagno and David 2009:15-16: "Sur les extrémités des pattes, des stries longitudinales présentes sur la face postérieure des métapodes, des premières et deuxièmes phalanges sont consécutives à la découpe de la peau". |
| **Bluefish Cave II** | | | |
| **Element and spec. number** | **Taxon** | **Observations** | **Ethnographic comparisons** |
| Scapula (fragment) # E2.4.1 | Snow goose *(Chen caerulescens)* | One short, deep and large cut mark, was first described by McCuaigBalkwill and Cinq-Mars (2008) | |
| Coxal bone* (fragment) # I5(e).6.5 | *Rangifer tarandus* | Three parallel and deep incisions longitudinally oriented on the lateral face of the ilium = possible filleting | Binford 1981:129: "[…] long, longitudinally oriented filleting marks […] shown on the pelvis […]". See also Binford 1981:130, Figure 4.36 shows marks produced on a caribou pelvis during filleting. |
| Metacarpal (shaft fragment) # D2.3.1 | Small ungulate (cf. *Ovis*) | One long and shallow trace longitudinally oriented on the anterior face of the shaft, with microstriations inside = possible filleting or removal of the periosteum | Binford 1981:134: "[…] longitudinal scratches and striations […] along the shafts of long bones are commonly produced when bones are prepared for cracking during marrow processing." See also Costamagno and David 2009:20: "[...] traces longitudinales, très fines et multiples sur les surfaces diaphysaires qui témoignent du raclage du périoste". |
| Mandible* (fragment, M2-M3 included) # J7.8.17 | *Equus lambei* | Multiple cutmarks, long and parallel, located on the medial side under the third and second molars = removal of the tongue | Binford 1981:109: "When the tongue is removed while the mandible is still attached to the skull marks are usually inflicted on the medial margins of the mandible […]". See also Binford 1981:100, Figure 4.07 shows diagonal cuts under the third and fourth premolars on a caribou mandible. |
| Radius (shaft fragment) # I5(e).2.2 | *Rangifer tarandus* | Short, large groove on the anterior face, near the distal portion = filleting or cutting the periosteum | Binford 1981:141, Tabl. 4.04 : "chevron marks on anterior face". See also Binford 1981:133 (Figure 4.39), Wheat 1979:63 (Figure 31) and Abe 2005:513-515 (Figure 7.55) who illustrate frequent oblique and transverse cut marks on the anterior face of the radio-ulna. |
